# An experimental test of energy and electrolyte supplementation as a mitigation strategy for white-nose syndrome

**DOI:** 10.1093/conphys/coz006

**Published:** 2019-02-20

**Authors:** Liam P McGuire, Heather W Mayberry, Quinn E Fletcher, Craig K R Willis

**Affiliations:** 1 Department of Biology, University of Winnipeg, 515 Portage Ave, Winnipeg, MB, Canada; 2 Present Address: Department of Biological Sciences, Texas Tech University, 2901 Main St, Lubbock, TX, USA; 3 Present Address: Department of Biology, University of Toronto Mississauga, 3359 Mississauga Rd. Mississauga, ON, Canada

**Keywords:** Bats, dehydration, electrolytes, hibernation, white-nose syndrome

## Abstract

Fungi are increasingly recognised as harmful pathogens of wildlife. White-nose syndrome (WNS) is a fungal disease that has killed millions of hibernating bats in North America. High mortality has driven research to identify management strategies for the disease. Increased energy expenditure and fat depletion, as well as fluid loss, hypotonic dehydration and electrolyte depletion appear to be key aspects of WNS pathophysiology. Bats with WNS spend energy too quickly and also lose fluids containing water and electrolytes from lesions on exposed skin surfaces. During periodic arousals, bats often drink water but, in most of the WNS-affected area, food is not available during winter and, therefore, they cannot maintain energy balance or replace lost electrolytes. Therefore, providing a liquid caloric/electrolyte/nutrient supplement could be useful for treating WNS. We studied captive, hibernating little brown bats (*Myotis lucifugus*) to test whether providing supplemental energy and electrolytes (a 1:1 dilution of unflavoured Pedialyte) to hibernating bats could reduce severity of WNS symptoms and increase survival. Infected bats in the Pedialyte-supplemented group generally avoided the Pedialyte and preferentially drank plain water. We did not observe any differences in survival, arousal frequency or blood chemistry, but bats in the Pedialyte-supplemented group had higher fungal load and more UV fluorescence than the control group that was only provided with water. Supplemental electrolytes would be an attractive management strategy because of their low cost and logistic feasibility but our results suggest this approach would be ineffective. However, it could be useful to conduct preference experiments with multiple dilutions and/or flavours of electrolyte solution. Although they did not prefer Pedialyte in our experiment, bats in the hand readily drink it and electrolyte supplementation remains an important tool for rehabilitation of captive bats recovering from WNS and other causes of dehydration.

## Teaser

White-nose syndrome is a fungal disease that has killed millions of hibernating bats. One symptom is electrolyte depletion. We tested whether providing an electrolyte supplement, a potentially simple and cost-effective conservation intervention, to hibernating bats would reduce disease severity. The treatment was not effective and may have increased fungal growth.

## Introduction

Fungal pathogens are increasingly being implicated as causal agents of emerging infectious diseases in wildlife, and have caused dramatic population declines and biodiversity loss across the globe (Fisher *et al.*, 2012). Two of the most prominent examples are *Batrachochytrium dendrobatidis*, which causes chytridiomycosis and has led to declining amphibian populations worldwide (Berger *et al.*, 2016), and *Pseudogymnoascus destructans*, which causes white-nose syndrome (WNS) and has killed millions of hibernating bats in North America over the past decade. Changes in the relative abundance of bats on the landscape were observed soon after the discovery of WNS (Dzal *et al.*, 2011), and population models based on counts of bats in hibernacula emphasised the dramatic decline in populations affected by the disease (Frick *et al.*, 2010, 2017). After more than a decade since WNS was first observed (Blehert *et al.*, 2009), there is a growing understanding of the causes and pathophysiology of the disease (Lorch *et al.*, 2011; Warnecke *et al.*, 2012, 2013; Willis, 2015; Frick *et al.*, 2016; McGuire *et al.*, 2017; Mayberry *et al.*, 2018).

WNS now affects 11 species in North America, with a further six species testing positive for the causative fungus (https://www.whitenosesyndrome.org), prompting concerns of population-level impacts for many species (Frick *et al.*, 2015). As WNS continues to spread to new species and new regions, there is a major emphasis on identifying potential intervention strategies to mitigate the consequences of the disease (Willis, 2015; Frick *et al.*, 2016). Efforts have included possible treatments that may reduce or eliminate fungal infections (e.g. Chaturvedi *et al.*, 2011; Foley *et al.*, 2011; Cornelison *et al.*, 2014; Cheng *et al.*, 2017), or other approaches to managing the disease and minimising impacts to susceptible populations (Boyles and Willis, 2010; Hallam and McCracken, 2011).

*P. destructans* colonises exposed skin surfaces of hibernating bats, especially the wings, causing lesions in the epidermis where fungal hyphae penetrate into the dermis (Cryan *et al.*, 2010; Meteyer *et al.*, 2012; Warnecke *et al.*, 2013). This invasion leads to homeostatic disruptions that may begin early in hibernation (Verant *et al.*, 2014) but which become most pronounced in late hibernation (Warnecke *et al.*, 2013; McGuire *et al.*, 2016, 2017). The most classic symptom of WNS is a characteristic increase in the frequency of periodic arousals from torpor late in hibernation (Reeder *et al.*, 2012; Warnecke *et al.*, 2012, 2013; Verant *et al.*, 2014; McGuire *et al.*, 2017). Infected bats also exhibit higher torpid metabolic rate (TMR) than healthy bats (McGuire *et al.*, 2017). Frequent arousals and higher TMR deplete limited adipose stores, presumably leading to death by starvation (Warnecke *et al.*, 2013; Verant *et al.*, 2014).

Fluid loss across damaged skin is one possible driver of increased arousal frequency during WNS (Willis *et al.*, 2011). Bats with WNS suffer higher rates of evaporative water loss (EWL) compared with healthy bats (McGuire *et al.*, 2017) and also show depletion of blood electrolyte concentrations, notably sodium and chloride (Cryan *et al.*, 2013; Warnecke *et al.*, 2013; McGuire *et al.*, 2016). Dehydrated bats can drink water available in hibernacula during periodic arousals, but these water sources do not contain meaningful concentrations of electrolytes or other nutrients (Vanderwolf *et al.*, 2017). Thus, as bats lose body fluids containing water and electrolytes from epidermal lesions, they can replace the lost water by drinking, but cannot replace electrolytes, leading to a state of hypotonic dehydration (Warnecke *et al.*, 2013; Verant *et al.*, 2014).

Given the importance of premature fat depletion, increased EWL (Willis *et al.*, 2011; McGuire *et al.*, 2017) and hypotonic dehydration in WNS pathophysiology (Cryan *et al.*, 2013; Warnecke *et al.*, 2013; Verant *et al.*, 2014), one potential management option could be to provide a solution of supplemental energy and electrolytes to bats affected by WNS. Although winter foraging may be possible for WNS-affected bat species hibernating at relatively southern latitudes (Bernard and McCracken, 2017), such opportunities may be limited and are unavailable for bats at higher latitudes. Training large numbers of hibernating bats in the wild to eat supplemental food like mealworms is highly impractical but bats regularly drink from surfaces and standing water in hibernacula during winter. A nutritional/electrolyte supplement provided in liquid form could represent a simple and cost-effective intervention to help bats stave off the physiological consequences of WNS. Pedialyte (Abbott Nutrition, Abbott Laboratories, Columbus, OH, USA) is an energy/electrolyte supplement designed for treatment of dehydration in humans and widely used in bat rehabilitation (Lollar and Schmidt-French, 1998; Klug and Baerwald, 2010) and could provide both supplemental electrolytes and energy to hibernating bats with WNS. Pedialyte contains several electrolytes (including sodium and chloride), and the carbohydrates in just 2 ml of unflavoured, dilute Pedialyte would provide enough energy to offset one week’s worth of the increased torpid energy expenditure caused by WNS (McGuire *et al.*, 2017).

We conducted an experiment with captive hibernating bats to test the hypothesis that liquid nutritional supplementation could be an effective strategy to reduce disease severity and increase survival in bats with WNS. Specifically, we predicted that bats provided a liquid supplement including energy and electrolytes would survive longer, lose less body mass, arouse less frequently, have lower fungal loads and less UV fluorescence on wings (an indicator of epidermal lesions; Turner *et al.*, 2014; McGuire *et al.*, 2016), and have less pronounced alterations to blood chemistry profiles (e.g. sodium and chloride concentrations).

## Materials and Methods

In November 2013, we collected 54 adult male little brown bats (*Myotis lucifugus)* from a site north of Grand Rapids, Manitoba, Canada (53.4°N 99.5°W). At the time of collection, this site was >1000 km from the nearest known site with WNS, and all bats were confirmed negative for *P. destructans* by quantitative PCR (qPCR; Muller *et al.* 2013). We only collected adult male bats to limit possible age or sex influences, and to avoid impacts of removing females from the population. We placed bats in cloth bags in a temperature-controlled cooler for transport to the University of Winnipeg as described previously (Warnecke *et al.*, 2012, 2013; McGuire *et al.*, 2016, 2017; Cheng *et al.*, 2017). We attached a uniquely marked, modified datalogger (iButton, Embedded Data Systems, Lawrenceburg, Kentucky, USA; Reeder *et al.*, 2012) to each bat using ostomy cement (Montreal Ostomy Inc., Vaudreuil-Dorion, Quebec, Canada). The iButtons recorded skin temperature (*T*_sk_) at 15-min intervals throughout hibernation, and the unique markings allowed us to visually identify individual bats in near-IR video recordings (Mayberry *et al.*, 2018, see below). We inoculated all bats with *P. destructans* as described in previous studies (Warnecke *et al.*, 2012) and assigned each bat to one of three cages (see below) based on ranked body masses, to ensure no differences in initial body mass among treatments.

All bats were housed in nylon mesh cages (23 × 38 × 38 cm; modified from Exo Terra, Rolf C. Hagen Inc., Montreal, Quebec, Canada) built into a single temperature and humidity-controlled incubator (Caron Products 6040–1, Marietta, OH, USA) set to 7°C and 98% relative humidity (0.9818 kPa). We confirmed chamber conditions with temperature and humidity sensors (Hobo Microstation Datalogger, H21-002, Onset Computer Corporation, Bourne, MA, USA) which recorded conditions every 10 min. We monitored hibernating bats with motion-activated near-IR cameras (Model HT6501RVFHQ; Speco Technologies, New York, NY, USA) that were placed above each cage, ensuring an unobstructed view of all bats within each cage. We reviewed video recordings daily to monitor arousals and to identify individuals which met criteria for humane intervention. Any bats exhibiting signs of morbidity (roosting alone, near the bottom of the cage and/or with wings outspread) were removed for assessment with as little disturbance to remaining bats as possible. Any bats that were unresponsive during assessments were humanely euthanized under isoflurane anaesthesia, otherwise were returned to their cage to continue hibernating.

Our experiment consisted of three treatment groups: electrolyte supplementation, dextrose supplementation and water control. We provided supplemental electrolytes with a 1:1 dilution of unflavoured Pedialyte (Abbott Nutrition, Abbott Laboratories, Columbus, OH, USA) (Klug and Baerwald, 2010). At this dilution, the Pedialyte solution contained sodium 518 mg/l, chloride 620 mg/l, potassium 390 mg/l and zinc 3.9 mg/l. Pedialyte also contains dextrose and therefore, we provided the dextrose group with a 12.5-g/l dextrose solution (Sigma-Aldrich), the same concentration found in the dilute Pedialyte, to account for possible energetic effects of Pedialyte not associated with electrolyte supplementation. The control group was provided only plain tap water. In case either the dilute Pedialyte or dextrose solution were unpalatable to the bats, and because there is no scenario where wild bats could be forced to drink an electrolyte supplement (plain water is readily available in hibernacula), all cages also included a second dish with plain drinking water. We flushed all drinking dishes weekly using supply tubes that ran outside the environmental chamber to avoid opening the door and disturbing the hibernating bats.

Within the first week of captive hibernation, there was an unavoidable repair that had to be made to one of the cages, which we completed as quickly and quietly as possible. After this disturbance, bats were left to hibernate undisturbed and we monitored their condition based on video recordings. After 112 (water treatment) or 113 days of hibernation (Pedialyte, dextrose treatments), we terminated the experiment. All bats were humanely euthanized under isoflurane anaesthesia. We recorded body mass, collected swabs for qPCR analysis of fungal load, and took wing photos under UV light to quantify the amount of UV fluorescence associated with WNS (see McGuire *et al.*, 2016, 2017). Briefly, UV fluorescence was quantified as the proportion of the wing area in which the characteristic orange fluorescence of WNS was visible (Turner *et al.*, 2015). Finally, we collected blood in lithium–heparin-treated capillary tubes for immediate analysis of blood chemistry with a handheld analyser (i-STAT1 Vet Scan, Abaxis, Union City, CA, USA). Blood samples were analysed with EC8+ cartridges from which we recorded sodium, potassium, chloride, glucose, haematocrit, urea nitrogen, pH, partial pressure of carbon dioxide and bicarbonate. These same data were collected from bats that were removed prior to termination of the experiment based on humane intervention criteria. All methods were approved by the University of Winnipeg Animal Care Committee and conducted under Manitoba Conservation Wildlife Scientific Permit WB15396.

**Figure 1:**
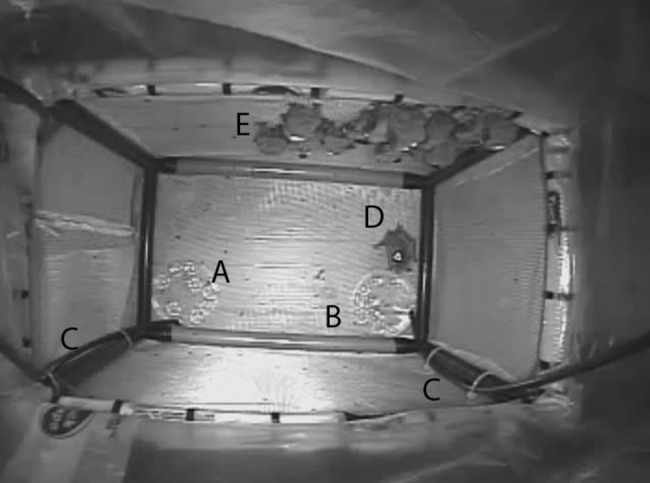
Screenshot from the Pedialyte supplementation treatment. The video camera provides a view down into the cage from above. There is a drinking dish at the front of the cage (**A**) and one at the back of the cage (**B**), one of which is filled with water and the other with dilute Pedialyte. The dishes are filled with tubes (**C**) that run outside of the environmental chamber to avoid disturbance to hibernating bats. The bat drinking from the back drinking dish (**D**) is identified by the symbol (triangle) drawn on the modified iButton that is glued to the back of the bat. In this screenshot, there is only one active bat (D), while all the remaining bats in the treatment are clustered in torpor on the side wall of the cage (**E**).

We used video recordings to document drinking behaviour in each treatment (Fig. 1). From January 1 until the end of hibernation (Days 56–112), we recorded all visits to one of the drinking dishes, noting the date, time, individual, and which dish they visited. Video observers were blind to the contents of drinking dishes during analysis. Our videos did not have sufficient resolution to definitively confirm drinking in all cases but we identified visits with obvious drinking behaviour (head dipping into water dish), movement of water caused by potential drinking (ripples caused by the head of a bat) or any time a bat remained in a drinking position for ≥3s (head positioned in drinking dish), as putative drinking events.

We compared survival among groups using a Breslow–Gehan survival analysis. Differences in blood chemistry, fungal load and UV fluorescence among groups were assessed using ANOVA with Tukey post hoc tests to identify pairwise differences. We assessed drinking preferences by using binomial tests including all putative drinking events to determine whether bats preferentially drank from one of the two dishes provided in each treatment, and also conducted a binomial generalized linear model (GLM) to compare drinking choices among treatments, accounting for the repeated measurements of individuals. Finally, we compared arousal frequencies with a linear mixed model, testing for treatment differences in the duration of torpor bouts, accounting for date and including individual as a random effect to account for repeated measures. We included only the final 50 days of hibernation in the analysis of torpor bout duration because arousal frequency only increases in late hibernation for bats with WNS. All statistical analysis was completed in R (v3.4.2; R Core Team, 2017).

## Results

No bats tested positive for *P. destructans* infection at the time of capture. Body mass at capture was 9.9 ± 0.1g, and did not differ among treatment groups (*F*_2,51_ = 0.09, *P* = 0.91). One bat in the water control group hibernated <50 days and was therefore excluded from our analysis, as this mortality was unlikely to be related to fungal infection. In some cases, cartridges were not available for the blood analyser, or bats died before it was possible to remove them for euthanasia, resulting in smaller sample sizes for blood chemistry analysis for water (*n* = 15), Pedialyte (*n* = 9) and dextrose (*n* = 14). Similarly, final body mass was only reliable for bats that were euthanized because there was no postmortem dehydration, resulting in sample sizes for the three experimental groups of 15, 11 and 14 for mass change throughout hibernation. Sample sizes for fluorescence were 16, 15 and 17, and for fungal load sample sizes were 17, 18 and 16. Finally, iButtons can fail after modification (Mayberry *et al.*, 2018), and we retrieved data from 12, 14 and 14 iButtons for water, Pedialyte and dextrose treatments.

We observed 477 putative drinking events. In the water control group, there were 54 drinking events at the front dish and 51 at the back dish. In the dextrose group, bats drank from the dish of dextrose 79 times compared with 75 drinking events at the water dish. In the Pedialyte treatment, there were 72 drinking events from the Pedialyte dish compared with 146 at the water dish. The total number of drinking events per individual varied among treatments (*F*_2,46_ = 6.04, *P* = 0.005) with more drinking events in the Pedialyte treatment compared with the water group (Tukey post hoc *P* = 0.003). Individual drinking preference was highly variable and, accounting for repeated measurements of drinking choices among individuals, the effect of treatment approached significance (binomial GLM, *F*_2,46_ = 2.77, *P* = 0.07) driven by an apparent preference for water in the Pedialyte treatment. With relatively few drinking events observed for many individuals, the binomial GLM may not have the power to detect drinking preferences for those individuals. Therefore, we also considered overall comparisons of drinking behaviour in each treatment, ignoring the effect of individuals. There was no preference for drinking dish in either the water (*P* = 0.85) or dextrose (*P* = 0.81) control treatments, but there was a preference for water over Pedialyte with bats drinking from the Pedialyte dish roughly half as often as from the water dish (*P* < 0.0001).

**Figure 2:**
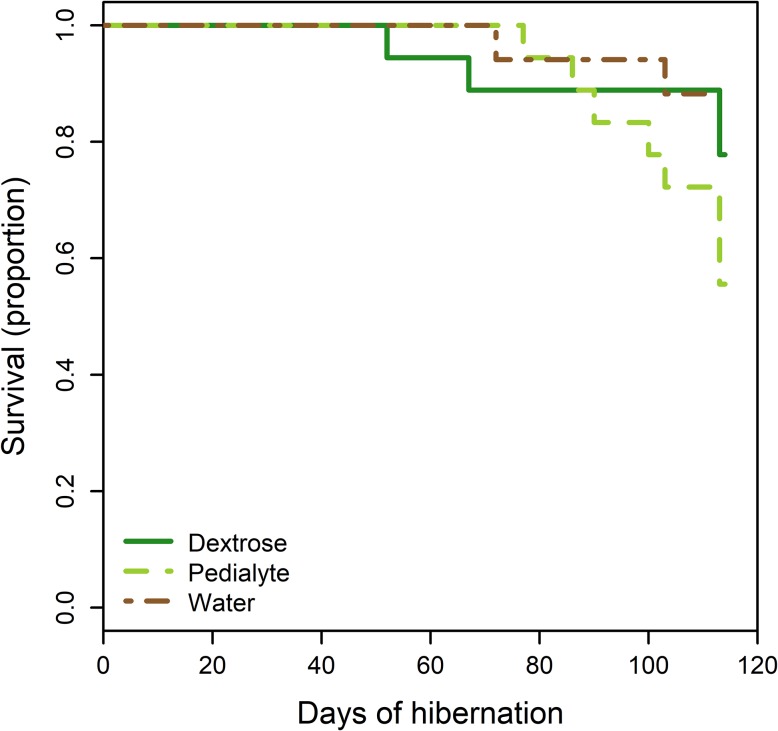
Electrolyte supplementation did not affect survival. There were no differences in survival among treatment groups. *n* = 17 for water treatment (control), *n* = 18 for dextrose and Pedialyte-supplemented group. The water treatment was terminated after 112 days of captive hibernation, the two other treatments were terminated the next day.

**Figure 3:**
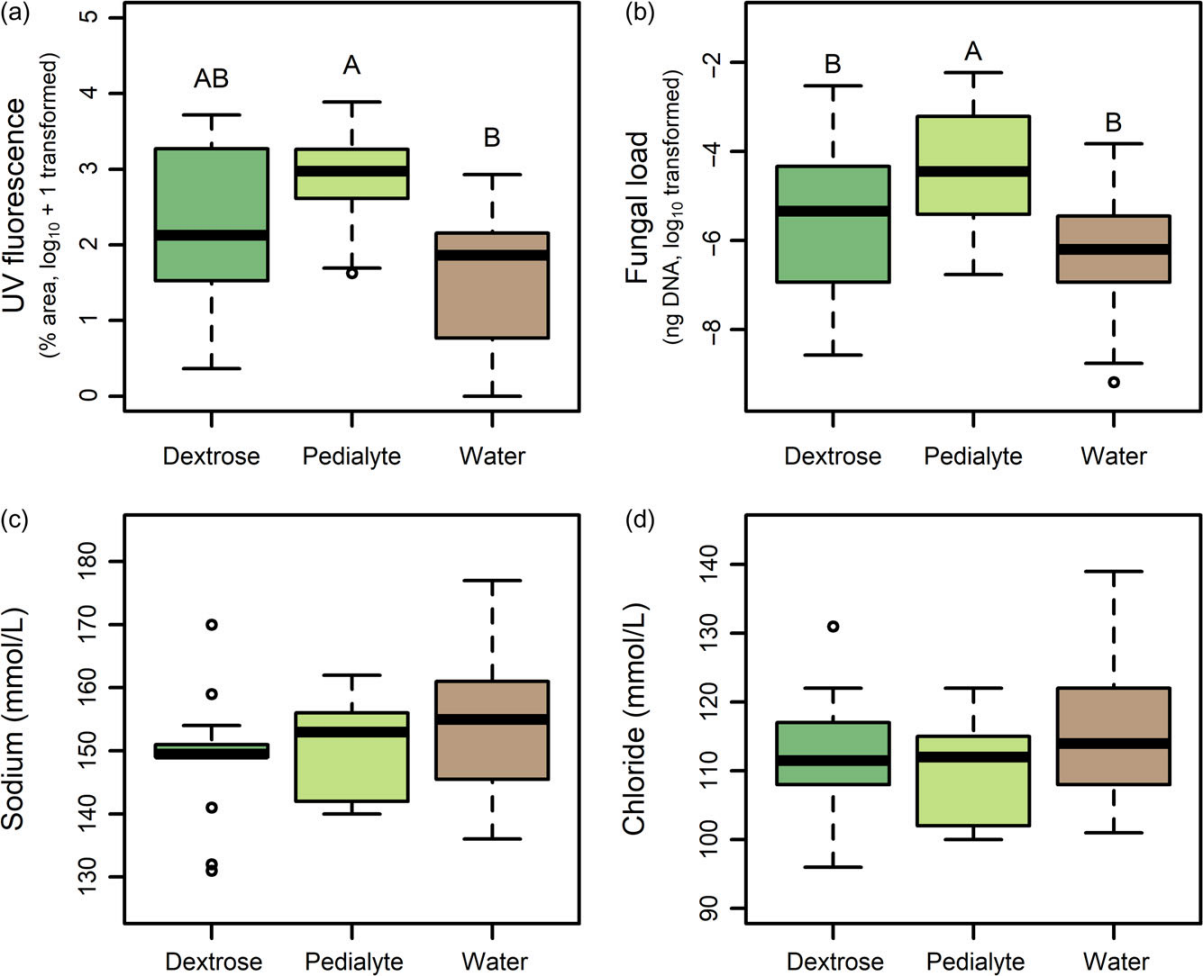
Electrolyte supplementation did not reduce disease severity. Compared with bats provided only water, electrolyte supplemented bats had (**a**) increased area of the wing affected by UV fluorescence, and (**b**) increased fungal load. Electrolyte supplementation had no effect on (**c**) blood sodium concentration, or (**d**) blood chloride concentration. There were no differences between bats provided dextrose solution and water controls for any factors. Where an overall treatment effect was found, groups sharing the same letter did not differ. Sample sizes are indicated in the text.

There was no difference in mass loss among the three treatments (*F*_2,37_ = 1.99, *P* = 0.15) and body mass declined to 7.7 ± 0.1g by the end of the experiment. Similarly, there was no difference in survival among the three treatments (Fig. 2; *χ*^2^ = 4.2, df = 2, *P* = 0.12). The duration of torpor bouts decreased over the final 50 days of hibernation (likelihood ratio = 67.2, df = 1, *P* < 0.0001), but there were no differences among treatment groups (likelihood ratio = 5.9, df = 2, *P* = 0.052). Bats in the Pedialyte treatment tended to have shorter torpor bouts than bats in the water control group (LS means contrast *P* = 0.055), but no other pairwise differences approached significance (*P* > 0.19). Bats in the Pedialyte group had more UV fluorescence (log_e_ + 1 transformed) than bats in the water control group (*F*_2,45_ = 7.96, *P* = 0.001; Tukey’s post hoc *P* < 0.001) but there were no other pairwise differences among treatments (all Tukey post hoc *P* > 0.12). Similarly, fungal load varied among treatments (*F*_2, 48_ = 7.49, *P* = 0.001), with bats in the Pedialyte treatment having higher fungal load than bats in the water (Tukey’s post hoc *P* = 0.001) or the dextrose treatment (Tukey’s post hoc *P* = 0.046). There was no difference between the fungal load of bats in the water or dextrose treatments (Tukey’s post hoc *P* = 0.37) (Fig. 3).

The only difference in blood chemistry among treatments was for haematocrit (*F*_2,35_ = 3.83, *P* = 0.03). Values for the Pedialyte treatment were less than for the water controls (Tukey post hoc tests *P* < 0.03), with no other pairwise differences. No other blood chemistry patterns, including sodium and chloride (Fig. 3), varied among treatments.

## Discussion

Unfortunately, we found no support for our hypothesis that a liquid electrolyte/carbohydrate supplement could provide therapeutic benefits for bats with WNS. Energy imbalance and electrolyte depletion are important components of mechanistic models proposed to explain mortality from WNS (Warnecke *et al.*, 2013; Verant *et al.*, 2014) and of the long list of homeostatic disruptions in WNS, electrolyte depletion seemed like a good target for intervention. Providing dilute Pedialyte to bats is an established approach in a rehabilitation context (Klug and Baerwald, 2010) and it would be relatively simple and cost-effective to deploy this product or similar liquid supplement as a drinking source in natural hibernacula. However, our study suggests this approach would not be effective at reducing the impacts of WNS. Bats in the Pedialyte treatment avoided drinking from the Pedialyte dish, and the Pedialyte they did drink had no positive effect on survival or any of the other metrics of disease severity we assessed. Furthermore, there were no differences between the water and dextrose treatments, indicating no energetic benefits independent of electrolyte supplementation.

Many aspects of WNS only become apparent or most pronounced late in hibernation (McGuire *et al.*, 2016). Our study continued for 112 days of captive hibernation, and we observed decreasing torpor bout duration (increasing arousal frequency) over the last 50 days of hibernation, as would be expected in late hibernation for bats with WNS. All but one bat had visible UV fluorescence, and in some cases UV fluorescence was extensive, indicating an advanced stage of the disease (McGuire *et al.*, 2016). The late stage of infection is further confirmed by fungal loads based on swabs taken at termination of the experiment. Therefore, it is unlikely that the lack of treatment effect we observed is due to insufficient hibernation duration, where differences between electrolyte supplemented bats and controls may not yet have been sufficiently pronounced. Furthermore, many disruptions of normal hibernation physiology are apparent even relatively early in hibernation (Verant *et al.*, 2014).

A simple prediction for our experiment was that the electrolyte depletion observed in WNS (Cryan *et al.*, 2013; Warnecke *et al.*, 2013) would be reduced or eliminated in the group provided with supplemental electrolytes. In fact, there were no differences among treatments in either of these electrolytes (Fig. 3c,d). Lower haematocrit in the Pedialyte group might suggests a reduction in hypovolemia (Warnecke *et al.*, 2013), but this potential benefit of electrolyte supplementation would probably have been counteracted by elevated UV fluorescence and fungal load in the Pedialyte treatment. Increased disease severity in the Pedialyte treatment may be related to the preference for water over dilute Pedialyte. While bats in the water control and dextrose treatments visited either of the two dishes in their cage, bats in the Pedialyte group demonstrated a clear preference for plain water. There were also more total drinking events in the Pedialyte group than for either of the other treatments. With the data available to us, it is not possible to determine whether increased drinking events in the Pedialyte group was a cause or effect of increased disease severity. One possibility is that increased exploration and/or activity within the Pedialyte cage, as bats searched for water and/or avoided Pedialyte, could have increased the number of times individual bats were exposed to *P. destructans* from substrates in the cage. If repeated exposures lead to multiple points of infection on the flight membranes, this could increase numbers of lesions on the wings (i.e. cause greater UV fluorescence)(Frick *et al.*, 2016) and cause bats to dehydrate more quickly (McGuire *et al.*, 2017) which may result in more frequent visits to the water dish. Alternatively, it is possible that Pedialyte provided nutrients for, and enhanced growth of *P. destructans* thereby increasing disease severity in that group.

Regardless of the explanation for increased disease severity in the Pedialyte-supplemented treatment, the unfortunate conclusion of our study is that liquid nutrient/electrolyte supplementation is unlikely to be effective for mitigating effects of WNS in wild bats. Simply put, bats did not voluntarily drink dilute Pedialyte, preferring instead plain water. However, dilute Pedialyte remains a useful option for rehabilitation and recovery of bats, in the hand, affected by WNS or suffering dehydration from other causes. We have used a 1:1 dilution of unflavoured Pedialyte, among other interventions, when treating bats recovering from WNS and other researchers have reported good results from use of Pedialyte for rehabilitating bats suffering dehydration (Klug and Baerwald, 2010). Bats in our experiment avoided Pedialyte in favour of plain water but this does not rule out the possibility that other nutrient/electrolyte solutions could be more readily exploited by bats and experiments testing the preferences of bats for a range of formulations could be worth pursuing (e.g. different dilutions, flavoured formulations). Experiments aimed at understanding the sensory cues used by bats would also help to understand decision making and drinking preferences. Future experiments that provide only dilute Pedialyte and do not provide the alternative of plain water could possibly better isolate the effects of Pedialyte, however such a scenario is unrealistic for deployment in natural conditions where plain drinking water is readily available to hibernating bats. Taken together our results do not support nutrient/electrolyte supplementation as a management approach for WNS despite the importance of energetic imbalance and electrolyte depletion to the pathophysiology of the disease.
